# Assessing the cost and economic impact of tertiary-level pediatric cancer care in Tanzania

**DOI:** 10.1371/journal.pone.0273296

**Published:** 2022-11-18

**Authors:** Anthony T. Saxton, Manisha Bhattacharya, Dharshan Sivaraj, Henry E. Rice, Nestory Masalu, Nelson J. Chao, Kristin Schroeder

**Affiliations:** 1 Department of Surgery, Duke University, Durham, NC, United States of America; 2 Duke Global Health Institute, Duke University, Durham, NC, United States of America; 3 Hillman Cancer Center, University of Pittsburgh Medical Center, Pittsburgh, PA, United States of America; 4 Division of Pediatric Oncology, Duke University, Durham, NC, United States of America; 5 Duke Cancer Institute, Duke University, Durham, NC, United States of America; 6 Department of Oncology, Bugando Medical Centre, Mwanza, Tanzania; 7 Department of Medicine, Duke University, Durham, NC, United States of America; Scuola Superiore Sant’Anna, ITALY

## Abstract

**Background:**

Worldwide, an estimated 400,000 children develop cancer each year. The bulk of the mortalities from these cases occur in low-and-middle-income countries (LMICs). In Sub-Saharan Africa, there is a tremendous need to strengthen the capacity of health systems to provide high-quality cancer care for children. However, a lack of data on the economic impact of cancer treatment in low-resource settings hinders its consideration as a healthcare priority. To address this gap, this study models the clinical and financial impact of pediatric cancer care in Tanzania, a lower-middle income country in East Africa.

**Methods:**

We conducted a retrospective review of patients with cancer under the age of 19 years treated at Bugando Medical Centre from January 2010 to August 2014. Information was collected from a total of 161 children, including demographics, type of cancer, care received, and five-year survival outcomes. This data was used to calculate the number of averted disability-adjusted life-years (DALYs) with treatment. Charges for all direct medical costs, fixed provider costs, and variable provider costs were used to calculate total cost of care. The societal economic impact of cancer treatment was modeled using the value of statistical life (VSL) and human capital methods.

**Findings:**

The total health impact for these 161 children was 819 averted DALYs at a total cost of $846,743. The median cost per patient was $5,064 ($4,746–5,501 interquartile range). The societal economic impact of cancer treatment ranged from $590,534 to $3,647,158 using VSL method and $1,776,296 using a human capital approach.

**Interpretation:**

Despite the limitations of existing treatment capacity, economic modeling demonstrates a positive economic impact from providing pediatric cancer care in Tanzania. As many countries like Tanzania progress towards achieving Universal Health Coverage, these key economic indicators may encourage future investment in comprehensive pediatric cancer care programs in low-resource settings to achieve clinically and economically beneficial results not only for the individual patients, but for the country as a whole.

## Introduction

Each year, an estimated 400,000 children are diagnosed with cancer, with 84% of cases occurring in low- and middle-income countries (LMICs) [1–3]. Although the survival rates for many pediatric malignancies have increased to over 80% in high-income countries (HIC), survival rates as low as 5–25% remain common in many LMICs [4,5]. Factors such as insufficient healthcare facilities, equipment, and trained personnel coupled with budgetary constraints in these settings have contributed to creating this “cancer divide.” Furthermore, assumptions that pediatric oncology programs are too costly, resource-intensive, and impractical have led many countries to disregard oncology capacity as a health care priority, limiting national investments [6]. Despite this, there are many examples of several well-executed initiatives to improve access to cancer care globally, mainly for adults [7,8]. These important initiatives have demonstrated a capacity to improve survival and other oncologic clinical outcomes despite challenging circumstances, and are starting to attract more government support. However, the understanding of the costs and benefits of cancer care for children in LMICs remains limited.

With more than a third of the estimated 53 million citizens living below the global poverty line, Tanzania’s economy was classified as “low-income” by the World Bank during the study period, with a recent change into the “lower-middle income” group in 2020 [9]. Pediatric cancer affects over 3,000 children per year in Tanzania, who face a multitude of barriers to access healthcare resources for diagnoses and treatment [5]. But like all children, they hold immense potential to contribute to their society if cured. Improved understanding of the costs to treat these children and the magnitude of the resulting economic impact can guide policy development and strengthen healthcare systems in Tanzania and similar LMICs.

This study models the clinical and financial impact of pediatric cancer treatment at a single referral hospital, the Bugando Medical Centre (BMC). Using data from BMC, it was hypothesized that a capable pediatric cancer program provides economic benefits for the region, and investments in pediatric oncology care were demonstrated to offer substantial returns even in challenging socioeconomic contexts.

## Methods

Located on the shores of Lake Victoria in Northern Tanzania, Bugando Medical Centre is one of three consultant hospitals in the country and serves a catchment area of over 15 million people. A medical oncology unit was established in 2009 to support the diagnosis and treatment of adults and children as one of only two referral cancer centers in Tanzania [10]. Diagnostic and treatment capacity here have been previously described [11].

A retrospective review of BMC hospital records from January 2010 to August 2014 was completed to collect data from all children less than 19 years of age with a cancer diagnosis. A total of 298 patient records were identified. Of these, 113 medical files were not available for review, and 3 additional files were excluded from analysis after further review did not identify a cancer diagnosis, leaving 182 medical files with a cancer diagnosis available for survival analysis. It was found that 21 of these 182 medical files did not include a comprehensive record of treatment to calculate costs, leaving 161 remaining records for cost analysis. Bivariate analysis was performed to compare the population characteristics of the included and excluded groups using t-tests.

Data collected included demographics (age, sex), cancer diagnosis and date of first related clinic or hospital presentation. Chart abstraction was performed to record all medications, imaging, laboratory studies, hospital stays, clinic appointments, and surgeries involved in the process of diagnosing and treating the patients. Classification of cancer was based on site and tumor morphology based on International Classification of Childhood Cancer (ICCC) site groups [12]. Five-year overall-survival outcomes were collected through record review or active follow-up by hospital social workers, the details of which have been reported elsewhere [11]. Of the 182 total patients with medical files available for review, 68 had incomplete follow-up and were not included in the survival calculation for reasons including treatment abandonment (n = 50), loss to follow-up (n = 15), or transfer to outside facility (n = 3), leaving 114 patients with appropriate follow-up in the survival calculation. It is unlikely that patients who abandoned treatment survived, but because their vital status could not be documented, they were not used in calculation of overall survival. However, additional sensitivity analysis was performed on the whole group if all children who abandoned care were assumed to have died.

### Cost of care

All costs of care were measured using a societal analytic framework to account for the totality of costs incurred by the healthcare system, the hospital, and the patient’s family [13]. For fixed-provider costs, items considered included capital, staff salaries and benefits, maintenance of the building and equipment, fuel, and utilities. The hospital land, building, and equipment were valued at $7,000,000 [10] and expensed using a straight-line depreciation method over 39 years for the building and seven years for equipment assuming a 14% resale value. Staff salaries and benefits were included for two oncologists, one clinical pharmacist, one social worker, three oncology nurses, and a pathologist. The costs of maintenance, fuel, and utilities for the cancer center at BMC could not be obtained, so values reported by Gosselin and colleagues for a similarly sized 50-bed hospital in Sierra Leone were used [14]. Thirty percent of fixed provider costs were allocated to pediatric patients for this study to reflect the breakdown between pediatric and adult patients treated at the cancer center. The total fixed provider costs were divided evenly amongst the 298 patients seen during this time period to avoid overestimation of costs for the group of 161 patients analyzed here. Variable provider costs included surgical consumables, which were tallied for each patient based on amounts recorded in surgical logs in the patient’s medical record.

Direct medical costs for patients included laboratory tests, medication, imaging, hospital stay, clinic visits, and surgical fees logged in the patient’s medical record. Radiation therapy was not used for any patient due to lack of availability at the time. The National Health Insurance Fund (NHIF) price schedule for medicines and medical consumables (with effect from July 1, 2016) was utilized to calculate the cost of medications. For items that did not appear on the NHIF list, a local pharmacy in Mwanza was consulted in January 2017 to assign prices. Receipts for cost of care provided at other facilities were requested and included in the total costs. Estimated travel costs for the patient and a caregiver were based on the recorded home address and number of round-trip visits needed for clinic visits and hospital stays.

All costs were converted 2011 International Dollars. Costs were recorded in local currency Tanzanian Shillings (TSh) using 2017 prices and were converted to International Dollars by dividing the local currency by the World Bank purchasing power parity (PPP) conversion factor for private consumption in Tanzania [9]. Costs recorded in USD were standardized to 2011 International Dollars by adjusting for inflation using World Bank’s gross domestic product (GDP) deflator for Tanzania with the following formula: [9]

$$c2011=c20XX*\frac{G2011}{G20XX}$$

where c20XX represents cost at the time it was incurred, G2011 is the GDP deflator in 2011, and G20XX is the GDP deflator the year the cost was incurred.

### Averted disability-adjusted life years (DALYs)

The clinical impact of pediatric cancer care at BMC was estimated using averted disability-adjusted life-years (DALYs), which represents the amount of poor health attributable to a disease condition (from either disability or death) that is avoided through a health care intervention. The DALY is a widely used metric of disease burden, initially described and broadly examined by Murray and colleagues in the series of Global Burden of Disease (GBD) studies [15].

Nomenclature for DALY descriptions (r, K, β) specifies the discount rate (r), age-weighting modulation (K), and age-weighting parameter (β) factored into the calculation. Application of age weighting and discounting in DALY calculations has varied in the literature, but current consensus exists that future health benefits should be discounted at the same rate as future costs, and age-weighting should be reserved for scenario analysis [13]. Thus, DALY scenarios were considered using a 3% discount rate with no age-weighting (3, 0, 0) in the main text of this study. Values for a sensitivity analysis with no discounting or age-weighting (0, 0, 0), and a 3% discount weight with 4% age-weighting (3, 1, 0.04) were also provided in **S1–S4 Tables**, with an example calculation provided in **S1 File**. Averted DALY calculations were performed using the equation cited by Shrime and colleagues. [13]:

$$Averted\;DALY=YLL(RD-RD_{postTx})+PST(RD_{postTx}\times YLL+YLD_{dz}-pCompl\times YLD_{compl})$$

    where:

    *YLL* = years of life lost

    *RD* = risk of death with no treatment

    *RD*_*postTx*_ = risk of death following unsuccessful treatment

    *PST* = probability of successful treatment

    *YLD*_*dz*_ = years lived with disability following an unsuccessful treatment

    *pCompl* = probability of complications arising after a successful treatment

    *YLD*_*compl*_ = years lived with disability due to complications after successful treatment

For each individual patient, the years of life lost (YLL) were calculated using the formula [16]:

$$YLL=\frac{{KCe}^{ra}}{{\left(r+\beta \right)}^{2}}\left[e^{-(r+\beta )(L+a)}[-(r+\beta )(L+a)-1]-e^{-(r+\beta )a}[-(r+\beta )a-1]\right]+\frac{1-K}{r}(1-e^{-rL})$$

where:

*K* = age-weighting modulation constant (0 for no age-weighting, 1 for age-weighting),

*C* = the adjustment constant for age-weights (0.1658),

*e* = natural logarithm root (2.72),

*r* = discount rate,

*a* = predicted age of death without treatment (assumed to be 2 years after oncology evaluation),

*β* = age weighting constant (0.04),

*L* = standard life expectancy in Tanzania (by sex) at age a [17].

The risk of death with no treatment (RD) and the risk of death following unsuccessful treatment (RD_postTx_) were assumed to be 100% based on local experience. Probability of successful treatment (PST) was calculated for 25 different pediatric malignancies treated at BMC using five-year survival rates (Table 1). All patients with hepatocellular carcinoma (HCC, n = 1), Ewing sarcoma (n = 2), fibrosarcoma (n = 2), and sinonasal adenocarcinoma (n = 1) were lost to follow-up, so a 35% survival rate was used for these malignancies based on projected 5-year survival figures reported by Renner and colleagues for comparable childhood cancer treatment in Ghana [18].

**Table 1:** Five-year survival rates at Bugando Medical Centre for children beginning treatment from January 2010 to August 2014.

	Incomplete follow-up					
Diagnosis	Treatment abandonment	Loss to follow-up	Transfer to outside facility	Appropriate follow-up	Total patients	Five-year survival rate^a^
Leukemia						
Acute lymphoblastic leukemia (ALL)	2	1	0	14	17	7%
Acute myeloid leukemia (AML)	0	0	0	1	1	0%
Chronic myeloid leukemia (CML)	1	0	0	2	3	0%
Leukemia, not otherwise specified	1	1	0	4	6	25%
Lymphomas and reticuloendothelial neoplasms						
Hodgkin lymphoma	1	0	0	5	6	40%
Burkitt lymphoma	5	6	0	21	32	29%
Non-Hodgkin lymphoma	5	1	1	7	14	29%
Lymphoma, not otherwise specified	2	1	1	8	12	0%
Neuroblastoma						
Neuroblastoma	0	0	0	1	1	0%
Retinoblastoma						
Retinoblastoma	5	2	0	13	20	0%
Renal tumors						
Wilms tumor	8	0	0	17	25	12%
Hepatic tumors						
Hepatoblastoma	3	1	0	5	9	0%
Hepatocellular carcinoma	1	0	0	0	1	-
Malignant bone tumors						
Ewing sarcoma	2	0	0	0	2	-
Osteosarcoma	2	0	0	1	3	0%
Soft-tissue sarcomas						
Fibrosarcoma	1	0	1	0	2	-
Kaposi sarcoma	2	1	0	5	8	60%
Rhabdomyosarcoma and embryonal sarcoma	2	0	0	2	4	0%
Sarcoma, not otherwise specified	2	0	0	1	3	0%
Germ-cell, trophoblastic and other gonadal tumors						
Intracranial, intraspinal, gonadal, and unspecified non-gonadal germ-cell tumors	1	0	0	2	3	50%
Other unspecified germ-cell tumors	0	0	0	1	1	100%
Carcinomas and other malignant epithelial neoplasms						
Adenocarcinoma	0	1	0	0	1	-
Other and unspecified carcinomas	1	0	0	1	2	0%
Nasopharyngeal carcinoma	0	0	0	1	1	0%
Other and unspecified tumors						
Other tumors	3	0	0	2	5	50%
**Total**	**50**	**15**	**3**	**114**	**182**	**18%**

^a^ Based on all patients with appropriate follow-up.

The years lived with disability (YLD) were calculated using the formula [16]:

$$YLD=DW\{\frac{{KCe}^{ra}}{{\left(r+\beta \right)}^{2}}\left[e^{-(r+\beta )(L+a)}[–(r+\beta )(L+a)-1]-e^{-(r+\beta )a}[-(r+\beta )a-1]\right]+\frac{1-K}{r}(1-e^{-rL})\}c$$

where:

*DW* = disability weight

*L* = duration of disability

*K*, *C*, *e*, *r*, *a*, *β* = same as above

To account for the patients who died after treatment, years lived with disability following an unsuccessful treatment (YLD_dz_) were calculated using a DW of 0.288, corresponding to the DW for cancer diagnosis and primary treatment from the 2013 GBD study [19]. The duration of disability (L) before eventual death was assumed to be two years.

For the survival group, the years lived with disability due to complications after successful treatment (YLD_compl_) was accounted for using a DW of 0.072, which corresponds to the weight for moderate heart failure [19], and a probability of complications arising after a successful treatment (pCompl) of 0.1 based on rates of late cardiotoxic effects from childhood cancer survivors undergoing doxorubicin therapy [20], a common chemotherapy agent used for this group of patients at BMC. The value of *L* was the standard life expectancy in Tanzania (by sex) at age *a* [17].

### Societal economic impact

Two approaches to model the societal economic impact of cancer treatment were used: the value of statistical life (VSL) and the human capital method. The VSL is the maximum amount an individual would be willing to spend to reduce his or her risk of dying [21]. This method is commonly used by government agencies to evaluate the potential economic gains of investments in resource-poor settings [21–24]. In countries where formal VSL studies have not been performed, estimates from other countries can be transferred using GDP per capita as a conversion factor [25]. Using VSL estimates extrapolated from HIC, Hammitt and Robinson report the VSL in Tanzania in 2007 as $164,900 considering an income elasticity (IE) of 1, and $26,700 considering an IE of 1.5. [26] Dividing those totals by the 72-year life expectancy used to generate the figures yielded annual value of statistical life-year (VSLY) of $2,290 and $371, respectively. These values were adjusted to 2011 International Dollars using the World Bank GDP deflator to account for inflation between 2007 and 2011. To determine the economic value of health outcomes obtained from cancer treatment, the VSLY values were multiplied by the corresponding averted DALYs for each patient.

The human capital approach equates the value of years of human life to the market value of the economic output produced by an individual over their lifetime. To calculate the economic impact of cancer treatment using this model, the averted DALYs for each patient were multiplied by purchasing power parity (PPP) estimates of gross national income (GNI) per capita in Tanzania from 2011 [9]. The economic values from both models represent gross estimates of societal economic welfare losses that were potentially avoided as a result of pediatric cancer treatment.

### Statistical analysis

Data was collected using Excel spreadsheets (Microsoft Corp., Redmond, WA) and exported to STATA v14.2 (StataCorp, College Station, TX) for analysis. Results for the costs, averted DALYs, and economic impact were calculated as median values with the interquartile range (IQR) per patient for each cancer type and summarized for the total group of patients.

### Ethical considerations

The study was approved by the Catholic University of Health and Allied Sciences/BMC Research Ethical Committee (Mwanza, Tanzania) and the National Institute for Medical Research—Lake Zone Medical Research Coordinating Committee (Mwanza, Tanzania). The study qualified for exemption (per 45CFR46.101(b)) by the Duke University Institutional Review Board (Durham, NC).

## Results

This study reviewed the hospital records of 161 children treated for cancer at Bugando Medical Centre from January 2010 to August 2014. The average age of the patients was seven years, with a range of one month to 18 years (Table 2). Thirty-nine percent (n = 63) of patients were female. The most common malignancies treated were lymphomas (n = 57, 36%), followed by leukemia (n = 26, 16%) and renal tumors (n = 23, 14%). There was no significant difference in average age, sex distribution, or diagnosis observed between children included in this study and those excluded due to medical files not available for review at the time of the study (p > 0.05).

**Table 2:** 

Characteristic	Included in financial analysis (total n = 161) n (%)
Age band	
0–3	43 (27%)
4–6	43 (27%)
7–9	29 (18%)
10–12	17 (11%)
13–15	17 (11%)
16–18	12 (7%)
Sex	
Male	98 (61%)
Female	63 (39%)
Diagnosis	
Leukemia	26 (16%)
Acute lymphoblastic leukemia (ALL)	17 (11%)
Acute myeloid leukemia (AML)	1 (1%)
Chronic myeloid leukemia (CML)	2 (1%)
Leukemia, not otherwise specified	6 (4%)
Lymphomas and reticuloendothelial neoplasms	57 (35%)
Hodgkin lymphoma	6 (4%)
Burkitt lymphoma	29 (18%)
Non-Hodgkin lymphoma	12 (7%)
Lymphoma, not otherwise specified	10 (6%)
Retinoblastoma	14 (9%)
Renal tumors	23 (14%)
Hepatic tumors	9 (6%)
Malignant bone tumors	5 (3%)
Soft-tissue sarcomas	14 (9%)
Germ-cell, trophoblastic and other gonadal tumors	4 (2%)
Carcinomas and other malignant epithelial neoplasms	4 (2%)
Other and unspecified tumors	5 (3%)

**Fig 1:**
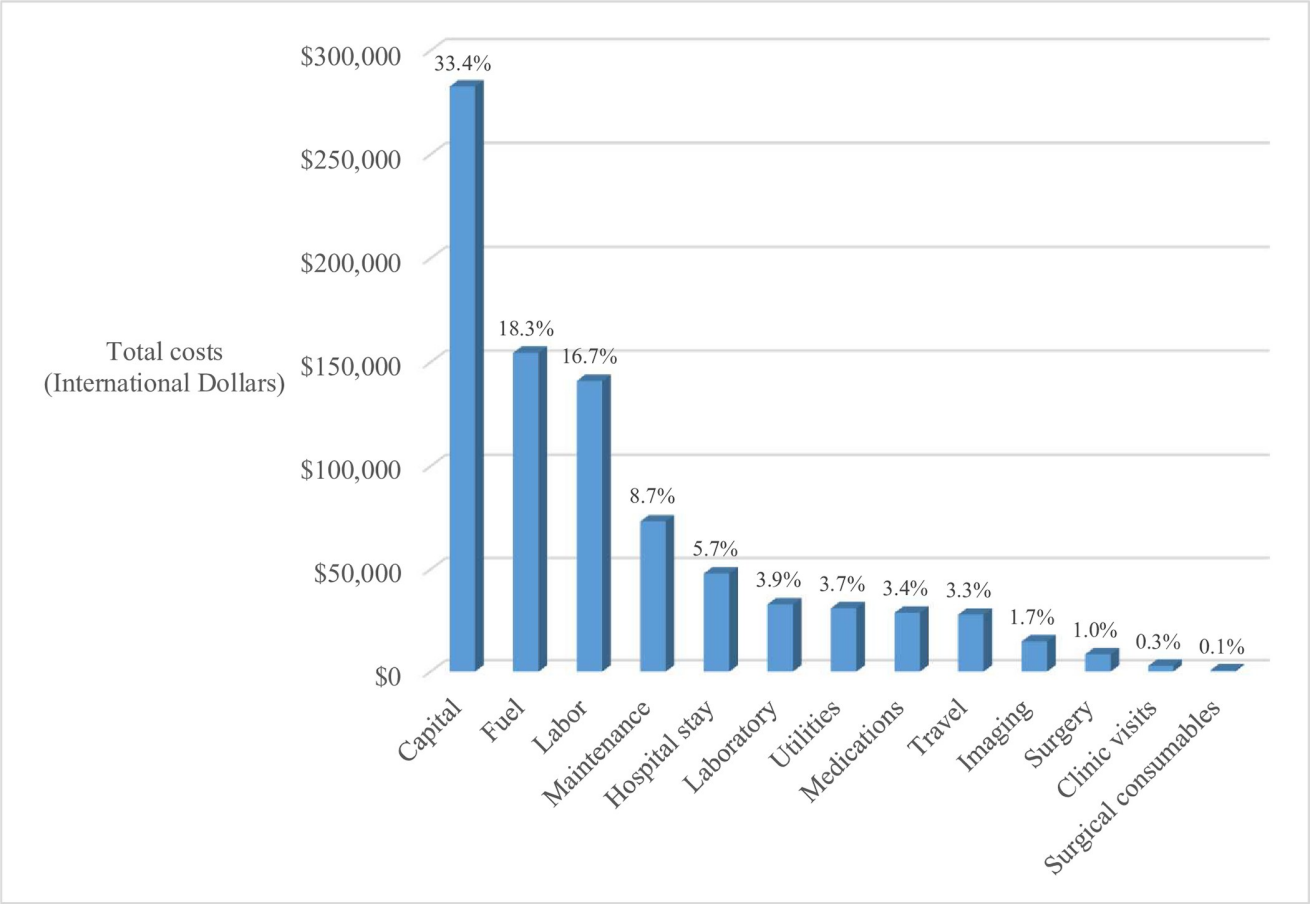
Total costs of pediatric cancer treatment for 161 children at Bugando Medical Centre from January 2010 to August 2014.

The total cost for diagnosis, treatment, and evaluation from a societal perspective for all 161 patients was $846,743. Fixed and variable costs to the hospital accounted for $682,619 (81%) of the total expenses, with capital (33%) and fuel (18%) representing the two largest items (Fig 1). Direct medical costs for the patients totaled $135,664 (16%), with hospital stay (6%) and laboratory fees representing the two largest items charged to the patient’s family. Direct non-medical costs for the patients and a caretaker were $27,791 (3%) for travel expenditures.

**Fig 2:**
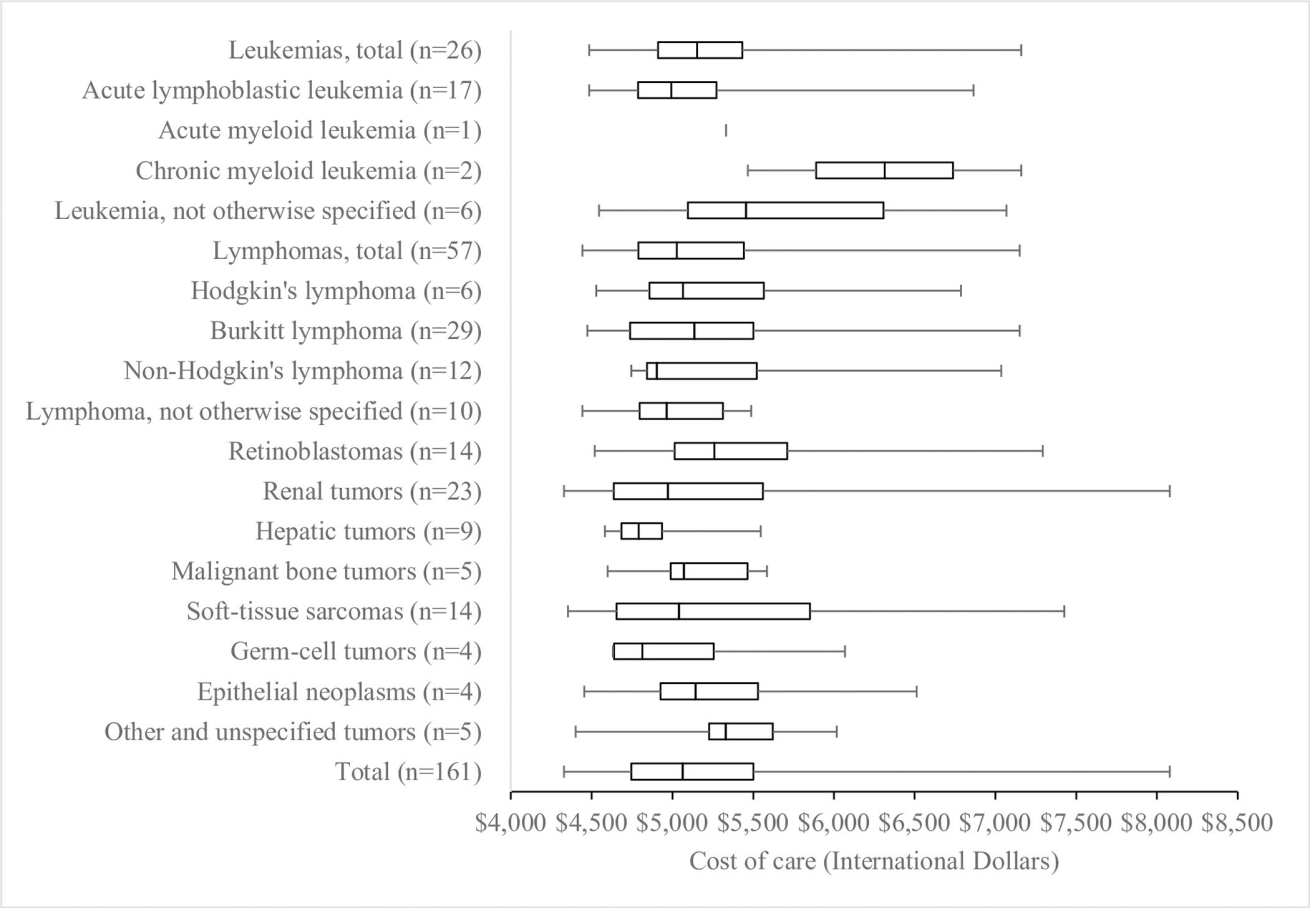
Boxplot of cancer treatment cost by diagnosis for 161 children at Bugando Medical Centre from January 2010 to August 2014.

The median cost of care per patient was $5,064 ($4,746–5,501 IQR) (Fig 2). The highest median cost per type of cancer was the group of other and unspecified tumors at $5,331 ($5,227–5,622 IQR), followed by retinoblastomas at $5,261 ($5,014–5,712 IQR). Hepatic tumors had the lowest median cost of $4,791 ($4,685–4,937 IQR).

The total health impact for these 161 children was 818.6 averted DALYs (Table 3). On a per-child basis, the median health impact amongst all cancers was 3.35 (0–7.96 IQR) averted DALYs. The two highest median DALYs averted by cancer type were 13.97 (10.10–17.80 IQR) for germ-cell tumors and 13.57 (13.47–13.89 IQR) for other and unspecified tumors. Using a VSL approach, the total economic benefit of treating these children ranged from $590,534 to $3,647,158 (Table 4). With a human capital approach, the total economic benefit was $1,776,296.

**Table 3:** Disability-adjusted life years averted through cancer treatment of 161 children at Bugando Medical Centre from January 2010 to August 2014.

	Total cases	DALY_a_ (3, 0, 0)*		
Cancer type		Median (IQR)	Mean (±SD)	Total
Leukemias	26	2.01 (1.98–2.06)	2.9 (± 2.28)	75.4
Acute lymphoblastic leukemia (ALL)	17	1.99 (1.99–2.03)	2 (± 0.04)	34.0
Acute myeloid leukemia (AML)	1	0 (0–0)	0 (± 0)	0.0
Chronic myeloid leukemia (CML)	2	0 (0–0)	0 (± 0)	0.0
Leukemia, not otherwise specified	6	6.97 (6.97–6.97)	6.9 (± 0.15)	41.4
Lymphomas	57	7.96 (7.49–8.11)	6.82 (± 3.27)	388.8
Hodgkin lymphoma	6	10.82 (10.56–11.05)	10.81 (± 0.26)	64.9
Burkitt lymphoma	29	7.96 (7.94–7.96)	7.88 (± 0.21)	228.4
Non-Hodgkin lymphoma	12	8.05 (7.75–8.13)	7.96 (± 0.25)	95.5
Lymphoma, not otherwise specified	10	0 (0–0)	0 (± 0)	0.0
Retinoblastoma	14	0 (0–0)	0 (± 0)	0.0
Renal tumors	23	3.34 (3.28–3.35)	3.32 (± 0.07)	76.4
Hepatic tumors	9	0 (0–0)	0.98 (± 2.77)	8.8
Malignant bone tumors	5	0 (0–9.18)	3.86 (± 4.74)	19.3
Soft-tissue sarcomas	14	9.46 (0–16.15)	8.31 (± 7.54)	116.4
Germ-cell tumors	4	13.97 (10.1–17.8)	13.93 (± 9.83)	55.7
Epithelial neoplasms	4	0 (0–2.49)	2.49 (± 4.31)	10.0
Other and unspecified tumors	5	13.57 (13.47–13.89)	13.56 (± 0.33)	67.8
**TOTAL**	**161**	**3.35 (0–7.96)**	**5.08 (± 4.93)**	**818.6**

DALY_a_, disability-adjusted life-years averted; SD, standard deviation.

* The nomenclature for DALY calculations (r, K, β) is used to specify the discount rate (r), age-weighting modulation (K), and age-weighting parameter (β) factored into the calculation. DALY of (3, 0, 0) represents a 3% discount rate and no age-weighting.

**Table 4:** Total economic benefit of cancer treatment of 161 children at Bugando Medical Centre from January 2010 to August 2014.

		Median and total economic benefit*			
	Total cases	VSL			
Cancer type		IE 1	IE 1.5		Human Capital
Leukemias	26	$8,954 ($8,843–9,170)	$1,450 ($1,432–1,485)		$4,361 ($4,307–4,466)
Acute lymphoblastic leukemia (ALL)	17	$8,872 ($8,845–9,036)	$1,437 ($1,432–1,463)		$4,321 ($4,308–4,401)
Acute myeloid leukemia (AML)	1	$0 ($0–0)	$0 ($0–0)		$0 ($0–0)
Chronic myeloid leukemia (CML)	2	$0 ($0–0)	$0 ($0–0)		$0 ($0–0)
Leukemia, not otherwise specified	6	$31,051 ($31,049–31,053)	$5,028 ($5,027–5,028)		$15,123 ($15,122–15,124)
Lymphomas	57	$35,485 ($33,383-$36,142)	$5,746 ($5,405–5,852)		$17,282 ($16,259–17,603)
Hodgkin lymphoma	6	$48,187 ($47,055–49,232)	$7,802 ($7,619–7,971)		$23,469 ($22,917–23,978)
Burkitt lymphoma	29	$35,485 ($35,372–35,485)	$5,746 ($5,727–5,746)		$17,282 ($17,228–17,282)
Non-Hodgkin lymphoma	12	$35,864 ($34,546–36,242)	$5,807 ($5,594–5,868)		$17,467 ($16,825-$17,651)
Lymphoma, not otherwise specified	10	$0 ($0–0)	$0 ($0–0)		$0 ($0–0)
Retinoblastoma	14	$0 ($0–0)	$0 ($0–0)		$0 ($0–0)
Renal tumors	23	$14,882 ($14,612–14,922)	$2,410 ($2,366–2,416)		$7,248 ($7,117–7,268)
Hepatic tumors	9	$0 ($0–0)	$0 ($0–0)		$0 ($0–0)
Malignant bone tumors	5	$0 ($0–40,884)	$0 ($0–6,620)		$0 ($0–19,912)
Soft-tissue sarcomas	14	$42,167 ($0–71,942)	$6,828 ($0–11,649)		$20,537 ($0–35,038)
Germ-cell tumors	4	$62,232 ($45,009–79,295)	$10,076 ($7,288–12,839)		$30,309 ($21,921–38,619)
Epithelial neoplasms	4	$0 ($0–11,099)	$0 ($0–1,797)		$0 ($0–5,406)
Other and unspecified tumors	5	$60,455 ($60,025–61,902)	$9,789 ($9,719–10,023)		$29,444 ($29,234–30,148)
**TOTAL**	**161**	**$3,647,158**	**$590,534**	** **	**$1,776,296**

VSL, value of a statistical life; IE, income elasticity.

* Calculated using disability-adjusted life-years (DALYs) averted with 3% discounting and no age-weighting. All values reported in 2011 United States dollars.

If all patients who abandoned care or were lost to follow-up were assumed to have died, median DALYs averted decreases from 3.35 (0–7.96 IQR) to 2.28 (0–5.23 IQR) and total economic impact decreases from $3,647,158 ($14,921 median) to $2,470,037 ($10,147 median).

## Discussion

This study estimates the economic benefit of providing pediatric cancer care in a low-resource setting. Bugando Medical Centre is the only site for cancer diagnosis and treatment within the Northwestern Tanzania region, and it is currently in the nascent stages of improving and expanding its capacity. Two economic models were used to transform measures reflecting cancer survivorship at BMC (DALYs averted) into estimates of how clinical care supports growth in national economic welfare. In our study, the societal economic impact of cancer care for 161 children yielded a rough benefit-to-cost ratio up to 4.3 for every dollar spent. As many countries like Tanzania progress towards achieving Universal Health Coverage, modest investments to support the diagnosis and treatment of cancer care for children in low-resource settings will benefit the economy as a whole. Targeting future investments strategically towards published interventions that have been shown to address the root causes of treatment failure in LMICs, such as with transportation, housing, infection prevention and control, and nurse training, could be expected to increase the benefit-to-cost ratio to at least 10.8 as survival rates match those seen in countries with established cancer centers with in low resource settings facilities [27–29].

Although the growing disease burden of pediatric cancer in LMICs is well-documented [5], there are few studies on the economic impact of treatment [30]. Liu and colleagues have found the average cost to treat childhood ALL in China is $11,000, [31] which drew the attention of the Chinese Ministry of Health to include ALL in a group of catastrophic diseases whose treatment costs are covered by government insurance for families who could not afford therapy [32]. ALL was also used as a case-study by Bhakta and colleagues who found that treatment of this disease for children in Brazil was well within “very cost-effective” thresholds per WHO-CHOICE criteria, as was Burkitt lymphoma treatment in Malawi [33]. A collaborative research group by Fuentes-Alabi and colleagues has developed a framework to evaluate cost-effectiveness of pediatric oncology care and applied it to clinical operations in El Salvador [34]. This framework was used to describe the care of 907 patients in El Salvador, with benefit quantified as US dollars/DALY averted. This methodology also demonstrated the pediatric cancer unit at Hospital Nacional de Niños Benjamin Bloom offered very cost-effective services. Renner and colleagues also found pediatric cancer treatment to be very cost-effective in Ghana for a similarly sized hospital [18]. Our results align with the findings of Bhakta, Fuentes-Alabi, and Renner to show that pediatric cancer treatment in Tanzania is very cost-effective, and our study expands on this result by attempting to conceptualize the economic gains of cancer treatment beyond spending tradeoffs within the healthcare budget.

There is increasing recognition of the role of Ministries of Finance and economic development councils in healthcare services [35]. Accordingly, this study uses methods from health, labor, and development economics to calculate the future economic benefit to the national labor market that can be attributed to treating children for life-threatening cancers. The World Bank’s Human Capital Index reinforces that these measures of economic benefit are commonly used as a development metric and are important outcomes. Thus, there is a compelling case for growth and expansion of cancer care infrastructure to be built into a country’s overall development agenda. This study is novel in calculating and comparing two distinct methodologies to translate cost-benefit metrics into estimations of general economic benefit. To our knowledge, this is the first estimate detailing the aggregate economic benefit of currently available pediatric cancer care in Africa as modeled on individual patient cost-effectiveness calculations. The models used in this study have previously been used to describe the economic impact of cleft lip and palate surgical repair [21–23]. Alkire and colleagues also applied these methods to estimate the macroeconomic consequences of head and neck cancer in South Asia, predicting large economic losses in the absence of treatment [25]. Our study applied primary data about survival outcomes at a regional cancer center to demonstrate how these potential economic losses can realistically be averted by access to cancer treatment. Contextualizing large investments in capacity in terms of future economic benefit of the treated patients over a lifetime may make these investments more justifiable.

The human capital and VSL approaches are two models commonly used to convert averted DALYs to dollars gained for a society, but their respective results vary widely. Understanding the assumptions of each methodology is essential to appreciate this variance and the context in which values are interpreted. The human capital approach uses GNI per capita as a proxy for the impact of averting a year of lost or impaired life. Using GNI makes the results realistic in the context of evaluating competing investments, but ultimately may be a conservative estimate of an intervention’s impact, as it cannot capture intrinsic value of life before and after an individual’s economically productive years [21,23]. In contrast, the VSL concept is designed to consider the personal value placed on mitigating or averting the risk of death or disability [36,37]. The advantage of this approach is the use of data derived from human behavior to approximate the marginal dollar value of each gained year of healthy life. An important distinction in interpreting human capital and VSL calculations is that VSL does not represent a direct economic loss from death or disability. It is more accurate to characterize the VSLY (incorporating DALYs and the duration of lifespan after the index illness) as Value of Lost Economic Welfare. This conceptualization yields estimates that are more inclusive than human capital estimates, which is limited to the generation of goods and services; VSLY also adds consideration of the relative expected duration of economic activity after the index illness. Together, these two estimates of economic impact are quite powerful for policymakers because VSLY describes the magnitude of current economic welfare losses and human capital quantifies a potential benefit in terms of an economic gain from treatment. In this study, it was found that the magnitude of current economic welfare losses averted through cancer treatment was as high as $3.6 million using VSLY, whereas the impact on GNI using a human capital approach was $1.8 million.

We recognize that economic analyses should be considered collectively with other tools and principles to set health care priorities, applying principles of “population medicine” [38]. The limited financial resources in LMICs necessitate funding entities to prioritize investments that maximize health outcomes. Inherent skepticism about cancer care in LMICs is likely linked to the many analyses of the finances of HIC cancer care where costs are discussed on the scale of hundreds of thousands of dollars per patient. However, we found that the costs of providing cancer services in Tanzania is quite low in comparison to other settings, with an average of $5,064 per patient. In addition to paying for basics of cancer care, further investment in low-cost but high-impact strategies to maximize initial efficacy and benefit should be prioritized.

This study’s limitations are largely tied to the necessary reliance on retrospectively analyzed clinical data from a single site. For example, DALYs are typically intended to measure the morbidity and mortality of diseases at a population level, not at an individual level. However, given the size of the cancer center and the inclusion of patients over a five-year period, we believe this is a reasonable approximation of clinical impact. In fact, the use of survival outcomes for each type of malignancy treated at our hospital generates a more precise estimation of DALYs compared with data reported at the population level. The retrospective nature of the data collection from the medical records creates the possibility that the costs of some lab tests or medications are not included in the file. To minimize this impact, the expenses of each individual patient were analyzed to ensure that the treatment received aligns with current practices at the cancer center for that malignancy and that no major expenditures were missing, resulting in the exclusion of 21 individuals from final data analysis.

The societal analytic framework used for costing in this study accounts for comprehensive costs as incurred by patients, providers and institutions. The framework does not make any assumptions about patient-facing fees being set at levels that compensate the hospital or providers for their costs. In a pure single-payer system this framework would have generated significant double counting of expenses. In the current Tanzanian context and by the norms of many LMICs, there may be some overlap between the clinic fixed costs and the clinic costs charged to patients, but the bulk of patient-incurred costs fall under the hospital fixed cost allocation and as direct medical costs. The direct medical costs include many items that patients have to pay for separately and/or purchase individually outside the hospital grounds, unlike many HIC hospitals that stock and directly provide consumables to patients by bundling costs into hospital stay fees.

Another limitation is the exclusion of 113 individuals whose paper charts were not available for analysis at the time of this study. Moving forward, this limitation will be avoided through the utilization of a computer database that is being established at BMC. Incomplete patient follow-up was another problem encountered with cancer care at this institution. Of the patients with incomplete follow-up for survival analysis, 74% (n = 50) abandoned treatment during therapy, 22% (n = 15) were lost to follow-up after completion of therapy, and 4% (n = 3) were due to transferring care to another hospital. In Tanzania, as in many low resource settings, treatment abandonment is one of the most important contributors to poor outcomes for children with cancer, with reported rates in Sub-Saharan Africa ranging from 5–70% [39,40]. However, there are several interventions that have successfully reduced treatment abandonment in other LMIC and may be appropriate next steps at this cancer center, including low-cost social support programs that provide accommodation, transport support, and patient navigation services in addition to subsidies for chemotherapy and treatment cost [41–44].

While the results from this study are from a single institution, this allows us to extrapolate our estimates as a reasonable representation of current cancer care in similar LMICs. As the overall survival rate at this center gradually improves to a level comparable to those in high-income countries, our calculations for economic impact would increase to as high as $15,918,871 for a similar cohort of 161 children. These figures justify the provision of high-quality, curative therapy for children in LMICs to save both lives and money.

## Conclusions

Even though pediatric cancer care in Tanzania is still in the nascent stages of development and estimated cure rates from the first cohort of patients treated at Bugando Medical Centre is only 18%, it is already highly cost-effective and expected to provide lasting economic benefits as survivors reach adulthood and enter the workforce. Modest investments to improve cure rates will only continue to amplify the benefits. These findings ultimately serve as reinforcement that it is financially realistic and beneficial to the economy to treat pediatric cancer in low-income countries.

## Supporting information

S1 Table Disability-adjusted life years averted through pediatric cancer treatment at Bugando Medical Centre from January 2010 to August 2014. (PDF)

S2 Table Economic impact per patient using value of a statistical life (VSL) approach. (PDF)

S3 Table Economic impact per patient using human capital approach. (PDF)

S4 Table Total economic benefit of cancer treatment of 161 children at Bugando Medical Centre from January 2010 to August 2014. (PDF)

S1 File Example calculations. (PDF)
