# Supplementary material for: Assessing the cost and economic impact of tertiary-level pediatric cancer care in Tanzania
Source: PLoS One. 2022 Nov 18;17(11):e0273296. doi: 10.1371/journal.pone.0273296 (PMC9674137; doi:10.1371/journal.pone.0273296)
Supplement: S1 Table — (PDF) [file pone.0273296.s001.pdf]

S1 table. Disability-adjusted life years averted through pediatric cancer treatment at Bugando Medical Centre from January 2010 to August 2014

		DALY$_a$*						
	Total	(3, 0, 0)			(0, 0, 0)			
**Cancer type**	**cases**	**Median (IQR)**	**Mean (±SD)**	**Total**	**Median (IQR)**	**Mean (±SD)**	**Total**	**Median (IQR**
Leukemias	26	2.01 (1.98-2.06)	2.9 (± 2.28)	75.4	4.28 (4.13-4.59)	6.05 (± 4.7)	157.3	2.59 (2.51-2.63
Acute lymphoblastic leukemia (ALL)	17	1.99 (1.99-2.03)	2 (± 0.04)	34.0	4.17 (4.13-4.39)	4.23 (± 0.21)	71.8	2.59 (2.52-2.59
Acute myeloid leukemia (AML)	1	0 (0-0)	0 (± 0)	0.0	0 (0-0)	0 (± 0)	0.0	0 (0-0
Chronic myeloid leukemia (CML)	2	0 (0-0)	0 (± 0)	0.0	0 (0-0)	0 (± 0)	0.0	0 (0-0
Leukemia, not otherwise specified	6	6.97 (6.97-6.97)	6.9 (± 0.15)	41.4	14.59 (14.58-14.59)	14.24 (± 0.78)	85.4	9.03 (8.86-9.11
Lymphomas	57	7.96 (7.49-8.11)	6.82 (± 3.27)	388.8	16.67 (14.29-17.55)	14.06 (± 6.76)	801.3	10.28 (10.05-10.49
Hodgkin's lymphoma	6	10.82 (10.56-11.05)	10.81 (± 0.26)	64.9	21.54 (20.34-22.79)	21.59 (± 1.33)	129.6	14.42 (14.22-14.48
Burkitt lymphoma	29	7.96 (7.94-7.96)	7.88 (± 0.21)	228.4	16.67 (16.52-16.67)	16.24 (± 1.06)	471.0	10.28 (10.22-10.46
Non-Hodgkin's lymphoma	12	8.05 (7.75-8.13)	7.96 (± 0.25)	95.5	17.18 (15.53-17.69)	16.73 (± 1.38)	200.7	10.35 (10.21-10.42
Lymphoma, not otherwise specified	10	0 (0-0)	0 (± 0)	0.0	0 (0-0)	0 (± 0)	0.0	0 (0-0
Retinoblastoma	14	0 (0-0)	0 (± 0)	0.0	0 (0-0)	0 (± 0)	0.0	0 (0-0
Renal tumors	23	3.34 (3.28-3.35)	3.32 (± 0.07)	76.4	7.23 (6.86-7.28)	7.13 (± 0.38)	164.0	4.19 (4.15-4.24
Hepatic tumors	9	0 (0-0)	0.98 (± 2.77)	8.8	0 (0-0)	1.78 (± 5.03)	16.0	0 (0-0
Malignant bone tumors	5	0 (0-9.18)	3.86 (± 4.74)	19.3	0 (0-17.5)	8.05 (± 10)	40.3	0 (0-12.23
Soft-tissue sarcomas	14	9.46 (0-16.15)	8.31 (± 7.54)	116.4	18.86 (0-31.97)	16.62 (± 15.13)	232.7	12.65 (0-21.61
Germ-cell tumors	4	13.97 (10.1-17.8)	13.93 (± 9.83)	55.7	29.59 (20.01-38.85)	29.26 (± 20.56)	117.1	17.54 (12.82-22.66
Epithelial neoplasms	4	0 (0-2.49)	2.49 (± 4.31)	10.0	0 (0-5.42)	5.42 (± 9.39)	21.7	0 (0-3.23
Other and unspecified tumors	5	13.57 (13.47-13.89)	13.56 (± 0.33)	67.8	27.18 (26.7-28.92)	27.24 (± 1.64)	136.2	18.11 (17.79-18.35
**TOTAL**	**161**	**3.35 (0-7.96)**	**5.08 (± 4.93)**	**818.6**	**7.28 (0-16.67)**	**10.47 (± 10.08)**	**1,686.5**	**4.21 (0-10.41**

DALY$_a$, disability-adjusted life-years averted; SD Standard deviation

* The nomenclature for DALY calculations (r, K, β) is used to specify the discount rate (r), age-weighting modulation (K), and age-weighting parameter (β) factored into the calculation. DALYa of (3, 0, 0) represents a 3% discount rate and no age-weighting, (0, 0, 0) represents no discounting or age-weighting, and (3, 1, 0.04) represents a 3% discount weight with age-weighting at 4%.
