# Supplementary material for: Assessing the cost and economic impact of tertiary-level pediatric cancer care in Tanzania
Source: PLoS One. 2022 Nov 18;17(11):e0273296. doi: 10.1371/journal.pone.0273296 (PMC9674137; doi:10.1371/journal.pone.0273296)
Supplement: S2 Table — (PDF) [file pone.0273296.s002.pdf]

S2 table. Economic impact per patient using value of a statistical life (VSL) approach

Cancer type	Total cases	Economic benefit, value of a statistical life (VSL) approach (International D				
		(3, 0, 0)		(0, 0, 0)		
		IE 1	IE 1.5	IE 1	IE 1.5	
Leukemias	26	$8,954 ($8,843-9,170)	$1,450 ($1,432-1,485)	$19,062 ($18,407-20,444)	$3,086 ($2,980-3,310)	$11,
Acute lymphoblastic leukemia (ALL)	17	$8,872 ($8,845-9,036)	$1,437 ($1,432-1,463)	$18,572 ($18,414-19,552)	$3,007 ($2,982-3,166)	$11,
Acute myeloid leukemia (AML)	1	$0 ($0-0)	$0 ($0-0)	$0 ($0-0)	$0 ($0-0)	
Chronic myeloid leukemia (CML)	2	$0 ($0-0)	$0 ($0-0)	$0 ($0-0)	$0 ($0-0)	
Leukemia, not otherwise specified	6	$31,051 ($31,049-31,053)	$5,028 ($5,027-5,028)	$64,990 ($64,978-65,003)	$10,523 ($10,521-10,525)	$40,
Lymphomas	57	$35,485 ($33,383-36,142)	$5,746 ($5,405-5,852)	$74,260 ($63,673-78,207)	$12,024 ($10,310-12,663)	$45,
Hodgkin's lymphoma	6	$48,187 ($47,055-49,232)	$7,802 ($7,619-7,971)	$95,988 ($90,631-101,519)	$15,542 ($14,675-16,438)	$64,
Burkitt lymphoma	29	$35,485 ($35,372-35,485)	$5,746 ($5,727-5,746)	$74,260 ($73,619-74,260)	$12,024 ($11,920-12,024)	$45,
Non-Hodgkin's lymphoma	12	$35,864 ($34,546-36,242)	$5,807 ($5,594-5,868)	$76,550 ($69,197-78,839)	$12,395 ($11,204-12,765)	$46,
Lymphoma, not otherwise specified	10	$0 ($0-0)	$0 ($0-0)	$0 ($0-0)	$0 ($0-0)	
Retinoblastoma	14	$0 ($0-0)	$0 ($0-0)	$0 ($0-0)	$0 ($0-0)	
Renal tumors	23	$14,882 ($14,612-14,922)	$2,410 ($2,366-2,416)	$32,203 ($30,584-32,457)	$5,214 ($4,952-5,255)	$18,
Hepatic tumors	9	$0 ($0-0)	$0 ($0-0)	$0 ($0-0)	$0 ($0-0)	
Malignant bone tumors	5	$0 ($0-40,884)	$0 ($0-6,620)	$0 ($0-77,949)	$0 ($0-12,621)	
Soft-tissue sarcomas	14	$42,167 ($0-71,942)	$6,828 ($0-11,649)	$84,012 ($0-142,450)	$13,603 ($0-23,065)	
Germ-cell tumors	4	$62,232 ($45,009-79,295)	$10,076 ($7,288-12,839)	$131,858 ($89,156-173,081)	$21,350 ($14,436-28,025)	$78,1
Epithelial neoplasms	4	$0 ($0-11,099)	$0 ($0-1,797)	$0 ($0-24,144)	$0 ($0-3,909)	
Other and unspecified tumors	5	$60,455 ($60,025-61,902)	$9,789 ($9,719-10,023)	$121,094 ($118,947-128,834)	$19,607 ($19,259-20,860)	$80,
**TOTAL**	**161**	**$3,647,158**	**$590,534**	**$7,514,096**	**$1,216,655**	

IE, income elasticity
