# Supplementary material for: Assessing the cost and economic impact of tertiary-level pediatric cancer care in Tanzania
Source: PLoS One. 2022 Nov 18;17(11):e0273296. doi: 10.1371/journal.pone.0273296 (PMC9674137; doi:10.1371/journal.pone.0273296)
Supplement: S3 Table — (PDF) [file pone.0273296.s003.pdf]

S3 table. Economic impact per patient using human capital approach

Cancer type	Total cases	Economic benefit, human capital approach (International Dollars)		
		(3, 0, 0)	(0, 0, 0)	(3, 1, 0.04)
Leukemias	26	$4,361 ($4,307-4,466)	$9,284 ($8,965-9,957)	$5,616 ($5,457-5,715)
Acute lymphoblastic leukemia (ALL)	17	$4,321 ($4,308-4,401)	$9,045 ($8,968-9,522)	$5,615 ($5,473-5,619)
Acute myeloid leukemia (AML)	1	$0 ($0-0)	$0 ($0-0)	$0 ($0-0)
Chronic myeloid leukemia (CML)	2	$0 ($0-0)	$0 ($0-0)	$0 ($0-0)
Leukemia, not otherwise specified	6	$15,123 ($15,122-15,124)	$31,653 (31,647-31,659)	$19,590 ($19,220-19,771)
Lymphomas	57	$17,282 ($16,259-17,603)	$36,167 ($31,011-38,089)	$22,298 ($21,807-22,755)
Hodgkin's lymphoma	6	$23,469 ($22,917-23,978)	$46,749 ($44,141-49,443)	$31,298 ($30,848-31,428)
Burkitt lymphoma	29	$17,282 ($17,228-17,282)	$36,167 ($35,855-36,167)	$22,298 ($22,183-22,700)
Non-Hodgkin's lymphoma	12	$17,467 ($16,825-17,651)	$37,282 ($33,701-38,397)	$22,469 ($22,162-22,621)
Lymphoma, not otherwise specified	10	$0 ($0-0)	$0 ($0-0)	$0 ($0-0)
Retinoblastoma	14	$0 ($0-0)	$0 ($0-0)	$0 ($0-0)
Renal tumors	23	$7,248 ($7,117-7,268)	$15,684 ($14,895-15,808)	$9,087 ($9,015-9,197)
Hepatic tumors	9	$0 ($0-0)	$0 ($0-0)	$0 ($0-0)
Malignant bone tumors	5	$0 ($0-19,912)	$0 ($0-37,964)	$0 ($0-26,542)
Soft-tissue sarcomas	14	$20,537 ($0-35,038)	$40,917 ($0-69,378)	$27,447 ($0-46,902)
Germ-cell tumors	4	$30,309 ($21,921-38,619)	$64,220 ($43,422-84,297)	$38,056 ($27,818-49,177)
Epithelial neoplasms	4	$0 ($0-5,406)	$0 ($0-11,759)	$0 ($0-7,005)
Other and unspecified tumors	5	$29,444 ($29,234-30,148)	$58,977 ($57,931-62,747)	$39,307 ($38,598-39,822)
**TOTAL**	**161**	**$1,776,296**	**$3,659,632**	**$2,317,359**
