# Supplementary material for: Assessing the cost and economic impact of tertiary-level pediatric cancer care in Tanzania
Source: PLoS One. 2022 Nov 18;17(11):e0273296. doi: 10.1371/journal.pone.0273296 (PMC9674137; doi:10.1371/journal.pone.0273296)
Supplement: S4 Table — (PDF) [file pone.0273296.s004.pdf]

S4 table. Total economic benefit of cancer treatment of 161 children at Bugando Medical Centre from January 2010 to August 2014.

Cancer type	Total cases	Mean and total economic benefit*		
		VSL		
		IE 1	IE 1.5	Human Capital
Leukemias	26	$12,920 (± $10,160)	$2,092 (± $1,645)	$6,292 (± $4,948)
Acute lymphoblastic leukemia (ALL)	17	$8,909 (± $161)	$1,442 (± $26)	$4,339 (± $79)
Acute myeloid leukemia (AML)	1	$0 (± $0)	$0 (± $0)	$0 (± $0)
Chronic myeloid leukemia (CML)	2	$0 (± $0)	$0 (± $0)	$0 (± $0)
Leukemia, not otherwise specified	6	$30,745 (± $686)	$4,978 (± $111)	$14,974 (± $334)
Lymphomas	57	$30,391 (± $14,589)	$4,921 (± $2,362)	$14,801 (± $7,105)
Hodgkin's lymphoma	6	$48,173 (± $1,178)	$7,800 (± $191)	$23,462 (± $574)
Burkitt lymphoma	29	$35,096 (± $936)	$5,683 (± $152)	$17,093 (± $456)
Non-Hodgkin's lymphoma	12	$35,453 (± $1,094)	$5,740 (± $177)	$17,267 (± $533)
Lymphoma, not otherwise specified	10	$0 (± $0)	$0 (± $0)	$0 (± $0)
Retinoblastoma	14	$0 (± $0)	$0 (± $0)	$0 (± $0)
Renal tumors	23	$14,798 (± $298)	$2,396 (± $48)	$7,207 (± $145)
Hepatic tumors	9	$4,365 (± $12,347)	$707 (± $1,999)	$2,126 (± $6,013)
Malignant bone tumors	5	$17,200 (± $21,108)	$2,785 (± $3,418)	$8,377 (± $10,280)
Soft-tissue sarcomas	14	$37,032 (± $33,579)	$5,996 (± $5,437)	$18,036 (± $16,354)
Germ-cell tumors	4	$62,072 (± $43,807)	$10,050 (± $7,093)	$30,231 (± $21,336)
Epithelial neoplasms	4	$11,099 (± $19,224)	$1,797 (± $3,113)	$5,406 (± $9,363)
Other and unspecified tumors	5	$60,439 (± $1,478)	$9,786 (± $239)	$29,436 (± $720)
**TOTAL**	**161**	**$3,647,158**	**$590,534**	**$1,776,296**

VSL, value of a statistical life; IE, income elasticity

* Calculated using disability-adjusted life-years (DALYs) averted with 3% discounting and no age-weighting. All values reported in 2011 United States dollars.
