# Supplementary material for: Assessing the cost and economic impact of tertiary-level pediatric cancer care in Tanzania
Source: PLoS One. 2022 Nov 18;17(11):e0273296. doi: 10.1371/journal.pone.0273296 (PMC9674137; doi:10.1371/journal.pone.0273296)
Supplement: S1 File — (PDF) [file pone.0273296.s005.pdf]

S5 Example Calculations

If we use a 4 year-old boy with Burkitt lymphoma as an example, the calculations would be as follows using a 3% discount rate with no age-weighting (3, 0, 0):

Years of life lost (*YLL*):

$$YLL = \frac{KCe^{ra}}{(r+\beta)^2}\left[e^{-(r+\beta)(L+a)}\left[-(r+\beta)(L+a)-1\right] - e^{-(r+\beta)a}\left[-(r+\beta)a-1\right]\right] + \frac{1-K}{r}\left(1-e^{-rL}\right)$$

where:
K = age-weighting modulation constant (0 for no age-weighting, 1 for age-weighting),
C = the adjustment constant for age-weights (0.1658),
e = natural logarithm root (2.72),
r = discount rate,
a = predicted age of death without treatment (assumed to be 2 years after oncology evaluation),
β = age weighting constant (0.04),
L = standard life expectancy in Tanzania (by sex) at age a

$$YLL = \frac{0*0.1658*2.72^{0.03*6}}{(0.03+0.04)^2}\left[2.72^{-(0.03+0.04)(58.20+6)}\left[-(0.03+0.04)(58.20+6)-1\right] - 2.72^{-(0.03+0.04)6}\left[-(0.03+0.04)6-1\right]\right] + \frac{1-0}{0.03}\left(1-2.72^{-0.03*58.20}\right)$$

$$= 27.52$$

Probability of successful treatment (PST):

PST = 6 alive / (6 alive + 15 deceased)
= 0.286

Years lived with disability following an unsuccessful treatment (YLD_{dz}):

$$YLD = DW\{\frac{KCe^{ra}}{(r+\beta)^2}\left[e^{-(r+\beta)(L+a)}\left[-(r+\beta)(L+a)-1\right] - e^{-(r+\beta)a}\left[-(r+\beta)a-1\right]\right] + \frac{1-K}{r}\left(1-e^{-rL}\right)\}c$$

where:
DW = disability weight
L = duration of disability before eventual death
a = predicted age of death with unsuccessful treatment
K, C, e, r, β = same as above

$$YLDdz = 0.288\left\{\frac{0*0.1658*2.72^{0.03*6}}{(0.03+0.04)^2}\left[2.72^{-(0.03+0.04)(2+6)}\left[-(0.03+0.04)(2+6)-1\right] - 2.72^{-(0.03+0.04)6}\left[-(0.03+0.04)6-1\right]\right] + \frac{1-0}{0.03}\left(1-2.72^{-0.03*2}\right)\right\}$$

$$= 0.56$$

Years lived with disability due to complications after successful treatment (YLD_{compl}):

where:
DW = disability weight
L = the standard life expectancy in Tanzania (by sex) at age a
a = predicted age of death with successful treatment
K, C, e, r, a, β = same as above

$$YLDcompl = 0.072\left\{\frac{0 * 0.1658 * 2.72^{0.03*65.3}}{(0.03+0.04)^2}\left[2.72^{-(0.03+0.04)(61.3+65.3)}\left[-(0.03+0.04)(61.3+65.3)-1\right] - 2.72^{-(0.03+0.04)65.3}\left[-(0.03+0.04)65.3-1\right]\right] + \frac{1-0}{0.03}\left(1-2.72^{-0.03*61.3}\right)\right\}$$
$$= 2.02$$

Averted DALY

$$Averted\ DALY = 27.52\ (1-1) + 0.286\ (1\ \times\ 27.52 + 0.56 - 0.1\ \times\ 2.02)$$
$$= 7.96$$

With averted DALY of 7.96 on average at a calculated cost of $5,548 from chart review, treatment of this patient yields a societal impact of $35,485 using a VSL approach (IE=1) or $17,282 from a human capital approach.
